# Evidence-based guidelines for hypofractionated radiation in breast cancer: conclusions of the Catalan expert working group

**DOI:** 10.1007/s12094-022-02798-8

**Published:** 2022-02-21

**Authors:** Arantxa Eraso, Javier Sanz, Meritxell Mollà, Vicky Reyes, Agustí Pedro, Meritxell Arenas, Evelyn Martinez, Rosa Ballester, Maria José Cambra, Virginia García, Joan Lluis Prades, Josep M. Borras, Manuel Algara

**Affiliations:** 1 Radiation Oncology Department, Institut Català d’Oncologia Girona, Avda de França,0, 17007 Girona, Spain; 2 Radiation Oncology Department, Hospital del Mar, Edifici B, -2. C/ del Gas s/n, 08003 Barcelona, Spain; 3 Radiation Oncology Department, Hospital Clínic de Barcelona, C/ Villarroel 170, 08036 Barcelona, Spain; 4 Radiation Oncology Department, Hospital Universitari Vall d’Hebron, Pass. de la Vall d’Hebron 119, 08035 Barcelona, Spain; 5 Radiation Oncology Department, Hospital de la Santa Creu i Sant Pau, C/ Sant Antoni Maria Claret 167, 08025 Barcelona, Spain; 6 Radiation Oncology Department, Hospital Universitari Sant Joan de Reus, Avda Doctor Josep Laporte 2, 43204 Reus, Spain; 7 Radiation Oncology Department, Institut Català d’Oncologia, Hospital Duran i Reynals, Avda Gran Via de l’Hospitalet, 199-203, 08908 L’Hospitalet de Llobregat, Spain; 8 Radiation Oncology Department, Institut Català d’Oncologia Badalona, Carretera del Canyet s/n, 08916 Badalona, Spain; 9 Radiation Oncology Deparment, Hospital General de Catalunya, C/ Pere i Pons 1, 08190 Sant Cugat del Vallés, Spain; 10 Radiation Oncology Departement, Hospital Universitari Arnau de Vilanova, Av. Alcalde Rovira Roure, 80, 25198 Lleida, Spain; 11 Pla Director d’Oncologia de Catalunya, Institut Català d’Oncologia, Hospital Duran i Reynals, Avda Gran Via de l’Hospitalet, 199-203, 08908 L’Hospitalet de Llobregat, Spain

**Keywords:** Breast cancer, Radiotherapy, Hypofractionation

## Abstract

**Introduction:**

Daily, moderate hypofractionation has become standard treatment for breast cancer following breast-conserving surgery, although substantial variation exists in its use. This paper describes the generation of consensus-based recommendations for the utilisation of this therapy at the healthcare system level and compares these to American Society for Radiation Oncology (ASTRO) guidelines.

**Materials and methods:**

Consensus-based guidelines were developed in three steps, including a systematic literature review and involvement of radiation oncologists specialising in breast cancer in Catalonia: (a) creation of a working group and evidence review; (b) consideration of the levels of evidence and agreement on the formulation of survey questions; and (c) performance of survey and development of consensus-based recommendations. Results were compared to the ASTRO recommendations.

**Results:**

Consensus was above 80% for 10 of the 14 survey items. Experts supported hypofractionated radiotherapy for all breast cancer patients aged 40 years or more; with invasive carcinoma and breast-conserving surgery; without radiation of lymph nodes; and regardless of the tumour size, histological grade, molecular subtype, breast size, laterality, other treatment characteristics, or need for a boost. Over half favoured its use in all situations, even where available scientific evidence is insufficient. The resulting recommendations and the quality of the evidence are comparable to those from ASTRO, despite some differences in the degree of consensus.

**Conclusion:**

Specialists agree that hypofractionation is the standard treatment for breast cancer following breast-conserving surgery, but some specific areas require a higher level of evidence before unequivocally extending indications.

## Introduction

Following breast-conserving surgery for breast cancer, daily, moderate hypofractionation has become standard treatment [1, 2]. Results from randomised trials do not support the use of classic fractionation of 2 Gy in most patients [3, 4], and studies have confirmed the effectiveness of hypofractionated regimens with similar tolerance [5]. Indications for its use have also broadened with the growing body of evidence showing its utility under other circumstances, for example with irradiation of lymph nodes [6] or the chest wall following mastectomy [7], or in patients of all ages [8]. However, there are substantial variations in the use of hypofractionation across different centres and countries [9, 10]. In a previous study, our group assessed its use for breast cancer in Catalonia, Spain [11], finding that it ranged from 8.9 to 74.7% of patients treated with a curative intent. In addition, specific indications for the treatment varied, both between services and among the different professionals that staff fed them.

In light of this heterogeneity and taking as a reference the American Society for Radiation Oncology (ASTRO) consensus guidelines on hypofractionation for breast cancer [12], we decided to launch a consensus-building process to establish recommendations for this breast cancer treatment in our region. Greater knowledge of the real indications and agreement among professionals should lead to an expansion of its use as a standard indication in Catalonia, promote equitable access, reduce differences between public healthcare services, and improve patients’ quality of life by avoiding the need for unnecessary travel and its associated inconveniences. This study describes the methods used to reach a consensus as well as the resulting recommendations as they compare to the ASTRO guidelines [12].

## Materials and methods

### Process

**Fig. 1:**
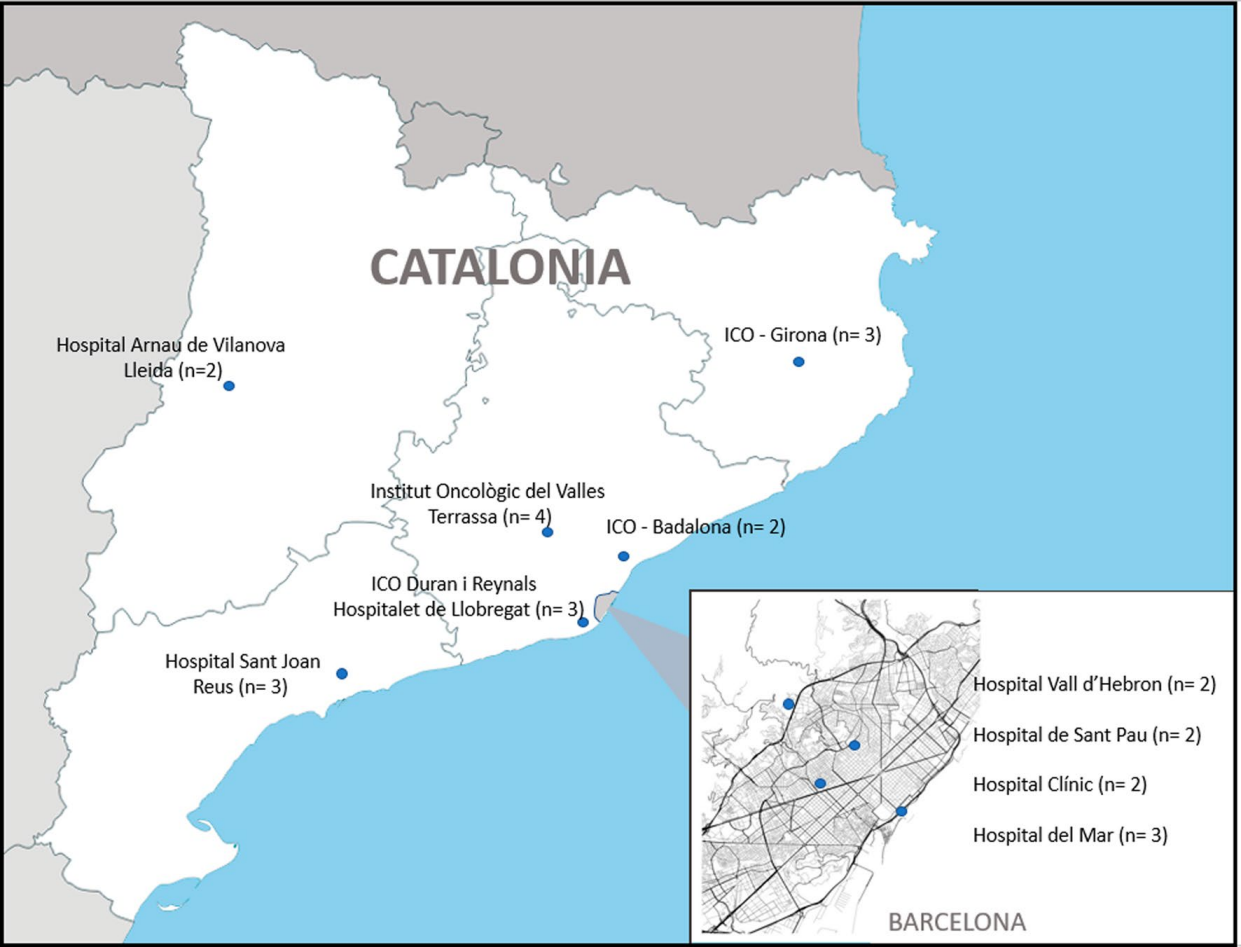
Participating centres and number of surveyed experts

In 2018, the Catalonian Cancer Plan approved the process to develop consensus-based guidelines on the use of hypofractionation for breast cancer. Work began in February 2018 and ended in May 2019, and consisted of three phases: (a) creation of a working group and systematic review of the evidence; (b) consideration of the levels of evidence and consensus on the formulation of survey questions; and (c) performance of the survey and development of consensus-based recommendations. Main invited experts were radiation oncologists specialising in breast cancer and working in the 10 public treatment centres in Catalonia; experts in evidence-based medicine, project management and biostatistics; collaborators with experience in searching and managing bibliographic databases; and managers and administrators involved in breast cancer treatment (heads of services, plus the Director of the Catalonian Cancer Plan) (Fig. 1). Altogether, a total of 26 professionals were invited to join the working group, and all accepted.

A steering committee of four members was created, bringing together professionals with responsibilities in clinical and technical leadership as well as project management and monitoring. Its functions were to define the objectives of the guidelines and the criteria for the bibliographic review; formulate the final clinical questions; synthesise and evaluate the evidence; and draft the final document.

In May 2018, the first in-person meeting of the consensus-building process took place. Fourteen questions were selected to answer the main controversial issues. The 14 survey questions were approved, and the most current literature was reviewed. The questions were then subjected to an independent vote among all experts of the working group. The vote was conducted online to avoid any influence from other experts of the group and enable a response after collating all the information.

**Fig. 2:**
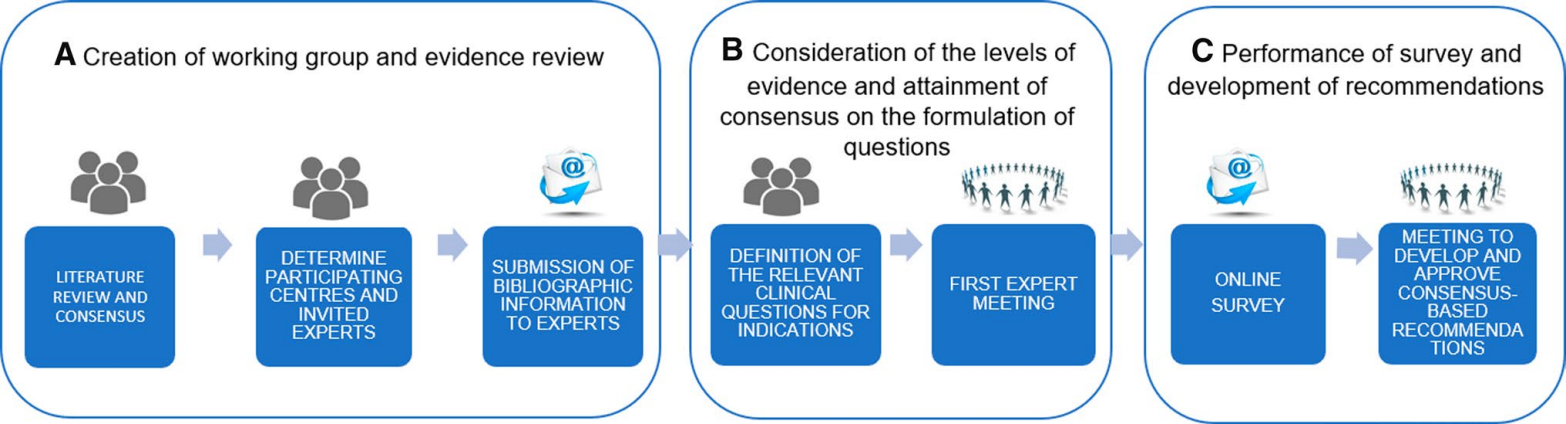
Methodology for the consensus-building process

In January 2019, the draft recommendations were presented to the working group during a second physical meeting to reach a final consensus among all the experts. The information gathered and the collective responses from the group were presented for each question, one by one, and the discrepancies and justifications were considered in turn. The recommendations were then drafted by consensus, and the direction and strength of each were finalised by May 2019. The methodological process is shown in Fig. 2.

### Literature review

The consensus-based guidelines were informed by an initial systematic literature review of records published in English between 1 January 2004 and 30 January 2019 and indexed in MEDLINE (PubMed). The primary search terms used were “hypofractionated”, “radiotherapy” and “breast neoplasm”.

**Fig. 3:**
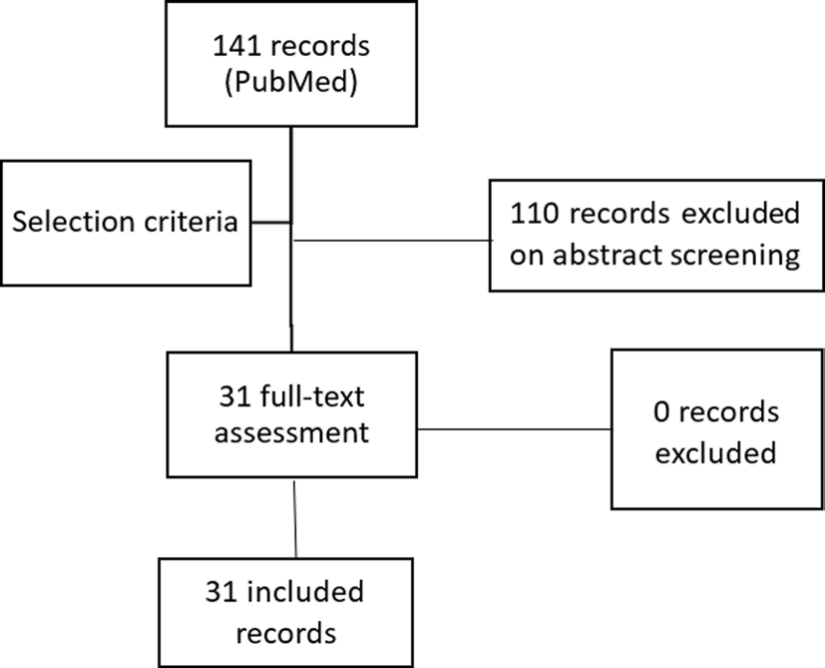
Results of the bibliographic search

The selection criteria for inclusion were randomised controlled trials (RCTs), meta-analyses of RCTs, and prospective observational studies that involved more than 100 participants. The intervention under assessment was hypofractionated external beam radiotherapy for early or locally advanced breast cancer, regardless of lymph node irradiation or additional boost. The outcomes of interest were control of breast cancer (disease-free survival, cancer-specific survival, and overall survival) and acute and late toxicity. The bibliographic search yielded 141 records. After screening the abstracts, 31 full-text records were evaluated, all of which fulfilled the established selection criteria (Fig. 3).

### Level of evidence, recommendations, and consensus-building methodology

The GRADE system was applied to classify the quality of the body of evidence and the strength of the recommendations [13, 14], in line with the approach used by ASTRO and the American Society for Clinical Oncology (ASCO) for their recommendations on hypofractionation in breast and prostate cancer [12, 15].

The recommendations were classified as strong or conditional, according not only to the quality of evidence, but also to other factors like the risk–benefit trade-off, the values and preference of patients and professionals, and the costs and resources involved. The members of the working group completed the survey by marking their agreement in the items on a scale of 0 to 10, with higher scores indicating stronger agreement.

A **strong recommendation** indicates that the working group concluded that the benefits of the intervention clearly outweighed the harms (or vice versa), and that ‘all or almost all of informed professionals would follow the recommendation. **Conditional recommendations** were made when the risks and benefits were similar or uncertain. According to our assessment scale, scores of 8 or above were considered a strong endorsement.

The **quality of evidence** for each recommendation was initially deemed high or low using ASTRO’s criteria [12] and Balsheim’s grades of evidence [14], according to whether the evidence was generated in experimental or observational studies. Other considerations were then taken into account to finalise the level of evidence as high, moderate, or low (Table 1).

- High: We are confident that the true effect is similar to the estimated effect.
- Moderate: We believe that the true effect is probably close to the estimated effect but substantially difference also is possible.
- Low: We have limited confidence in the estimated effect: the true effect might be markedly different from the estimated effect.

**Table 1:** American Society for Clinical Oncology (ASCO) criteria for assessing the quality of evidence

Rating	Quality criteria	Definition
High	- ≥ 2 well-conducted RCTs with generalisable results or meta-analysis of these trials	It is very likely that the true effect is close to the estimated effect, based on the body of evidence
Moderate	- 1 well-conducted, generalisable RCT or meta-analysis of trials; or - ≥ 2 RCTs with some limitations or lack of generalisability; or - ≥ 2 well-conducted observational studies with consistent results	It is likely that the true effect is close to the estimated effect, based on the body of evidence, but it is possible that it is substantially different
Low	- 1 RCT with some limitations or lack of generalisability; or - ≥ 1 RCTs with serious shortcomings, lack of generalisability, or extremely small sample sizes; or - ≥ 2 observational studies with inconsistent findings, small sample sizes, or other problems that may cloud the interpretation of data	The true effect may be substantially different from the estimated effect. There is a risk that future research may significantly alter the estimated size of effect or the interpretation of the results

*RCT* randomised controlled trial

In addition, experts’ opinions were integrated in the process using ASCO’s modified Delphi approach [16], producing a percentage of **consensus** among the working group. Members took an online survey, registering their level of agreement on a scale of 0 to 10. The pre-defined cutoff for consensus was 80%.

Results were compared to the ASTRO recommendations also considering quality of evidence and the consensus reached.

## Results

All the invited centres participated in every meeting held, all controversial issues were discussed and finally answered to the corresponding questionnaires submitted.

With regard to the 14 questions considered, these are detailed in Table 2, along with the level of evidence, degree of consensus, and strength of the recommendation.

**Table 2:** Indications on the use of hypofractionated radiation therapy for breast cancer in Catalonia, with consensus-based recommendations (> 80% agreement among working group members) marked with an asterisk

1. Hypofractionation is indicated in patients who have undergone breast-conserving surgery, regardless of tumour size*	
Recommendation: strong Quality of evidence: high Degree of consensus: 95%	*Justification* RCTs have shown hypofractionated treatment to be effective in tumours of up to 5 cm in diameter, so it is indicated following breast-conserving treatment in T1–T2 tumours [1, 2]
2. Hypofractionation is indicated in patients who have undergone breast-conserving surgery, including in women with nuclear grade 3 tumours*	
Recommendation: strong Quality of evidence: high Degree of consensus: 98%	*Justification* RCTs have not found any unfavourable effects associated with a high nuclear grade. In one trial, a second pathological review confirmed this issue [17, 18]. This could be related to the different pathological grading methodology applied by pathologists [19]
3. Hypofractionation is indicated in patients who have undergone breast-conserving surgery, independently of molecular subtype*	
Recommendation: strong Quality of evidence: moderate Degree of consensus: 97%	*Justification* In some included RCTs, molecular subtypes were not differentiated, or some were poorly represented, so the quality of the available evidence on the equivalence of hypofractionated treatment is not high. Despite the data showing good local tumour control, this tends to be lower for luminal B and basal molecular subtypes [17]
4. Hypofractionation is indicated in patients who have undergone breast-conserving surgery, independently of the laterality of the breast requiring treatment*	
Recommendation: strong Quality of evidence: high Degree of consensus: 99%	*Justification* Although RCTs have not stratified their results by laterality, indirectly it can be inferred that there are no differences in the fractionation scheme nor greater toxicity per dose in critical organs, or greater toxicity on the left side. Also, non-randomised trials with long follow-up have not found differences according to the side affected by the tumour [1, 2]
5. Hypofractionation is indicated in patients who have undergone breast-conserving surgery, independently of whether they receive neoadjuvant chemotherapy*	
Recommendation: strong Quality of evidence: low Degree of consensus: 89%	*Justification* Hypofractionation studies included patients receiving adjuvant chemotherapy, with no unfavourable impact. Thus, the consensus of the working group was that this intervention was safe in patients receiving primary systemic treatment with chemotherapy, targeted therapy, or hormone therapy [19]
6. Hypofractionation is indicated in patients who have undergone breast-conserving surgery, independently of whether they receive adjuvant chemotherapy*	
Recommendation: strong Quality of evidence: high Degree of consensus: 95%	*Justification* Although adjuvant chemotherapy was used in only 11% to 36% of participants in RCTs, Shaikh’s meta-analysis shows the safety of this treatment in hypofractionated patients [8]
7. Hypofractionation is indicated in patients who have undergone breast-conserving surgery and whose age is 40 to 50 years*	
Recommendation: strong Quality of evidence: high Degree of consensus: 88%	*Justification* There is enough evidence to support hypofractionated treatment in patients of all ages [1, 2]
8. Hypofractionation is indicated in patients who have undergone breast-conserving surgery and whose age is less than 40 years	
Recommendation: conditional Quality of evidence: moderate Degree of consensus: 63%	*Justification* There is sufficient evidence to support hypofractionated treatment in patients regardless of age, even though the proportion of younger women in trials is lower; moreover, evidence indicates that younger patients show better tolerance to hypofractionated treatment [21]
9. Hypofractionation is indicated in patients who have undergone breast-conserving surgery, including for histology that is exclusively carcinoma in situ	
Recommendation: conditional Quality of evidence: low Degree of consensus: 72%	*Justification* Despite the low quality of evidence, the same criteria were applied to hypofractionated radiotherapy for pure carcinoma in situ as to invasive tumours, pending results from an ongoing RCT (RTOG 9804) that specifically assesses this question [20]
10. Hypofractionation is indicated in patients who have undergone breast-conserving surgery, regardless of the size of the breast*	
Recommendation: strong Quality of evidence: high Degree of consensus: 95%	*Justification* State-of-the-art technologies allow a greater homogeneity of the dose, so increasing the dose per fraction would imply that the size of the breast is irrelevant [22–24]
11. Hypofractionation is indicated in patients who have undergone radical treatment and mastectomy	
Recommendation: conditional Quality of evidence: moderate Degree of consensus: 64%	*Justification* Only one RCT has included patients who underwent mastectomy showing that hypofractionation was not inferior in efficacy and had similar toxicities, so the level of evidence cannot be considered high [25], and the degree of consensus was low
12. Hypofractionation is indicated in patients receiving surgery for breast cancer who require nodal irradiation due to the involvement of lymph nodes	
Recommendation: conditional Quality of evidence: low Degree of consensus: 59%	*Justification* Given the lack of evidence, further studies are needed to specifically analyse the safety of regional lymph node irradiation using hypofractionated schemes, as evidence only exists for irradiation of the lower axilla, not for an intentional target volume encompassing the axillary region [26]. For this reason, the level of consensus was low
13. Hypofractionation is indicated in patients who have undergone breast-conserving surgery and have an indication for a tumour bed boost*	
Recommendation: strong Quality of evidence: high Degree of consensus: 97%	*Justification* RCTs included a tumour bed boost following hypofractionated treatment of the breast when indicated confirming the safety of this modality [2]
14. Hypofractionation of the tumour bed boost is indicated in patients receiving breast-conserving surgery for breast cancer*	
Recommendation: strong Quality of evidence: low Degree of consensus: 94%	*Justification* The boost can be performed with standard or hypofractionation (14–16 Gy to 2 Gy or 10–12.5 Gy to 2.5 Gy). The level of consensus was high because the use of 2.5 Gy per fraction has already been considered [27]

*RCT* randomised controlled trial

Our results are comparable to the ASTRO guidelines [12] in terms of the final recommendations and the quality of evidence supporting them. However, there were some differences in the level of consensus achieved, for example in patients under the age of 40 years: compared to ASTRO consensus, the agreement on the use of hypofractionation in these cases was 93%, compared to 63% in our analysis. These differences are probably due to our classification of patient age into three brackets: less than 40 years, 40 to 50 years, and more than 50 years.

Unlike the ASTRO guidelines, we included questions about hypofractionation in patients receiving a mastectomy or who required irradiation of the lymph node chain. For these indications, the degree of consensus was low at 64% and 59%, respectively, in consonance with the low quality of evidence.

## Discussion

Despite the growing evidence of the association between hypofractionated treatment and improved clinical and care quality for patients with breast cancer, the direct translation to clinical practice has been limited. The possible reasons for this lag are numerous and range from missed opportunities following positive results from clinical trials, to knowledge gaps and uncertainty, controversies, irrelevant or conflicting evidence, and vested or conflicting interests [28]. The considerable volume of scientific publications makes it difficult to attain deep knowledge based on the analysis of available evidence; rather, this is often synthesised at an individual level.

Uptake of hypofractionation has been slow for breast cancer in public hospitals in Catalonia, with considerable variability in clinical practice, as reported by Prades et al. [11]. This delay in the translation of evidence to practice has also been observed by other authors, for example Gilbo et al. [29], who observed an improvement in utilisation rates from 49 to 80% in the 4 years following dissemination of the ASTRO guidelines, implementation of clinical practice directives, and follow-up through departmental discussions about clinical indications. As Recht commented in an editorial [30], the use of hypofractionation will only reach recommended levels if specialists are engaged and adherence to guidelines is monitored.

Underpinning this perspective is the assumption that the adoption of healthcare innovations (including process-based innovations like hypofractionation) depends to some extent on how they are communicated and disseminated, not only on the quality of evidence that supports them. To that end, the work described in the present paper followed a consensus-based methodology that incorporates direct knowledge related to clinical practice into the scientific debate. The involvement of 11 clinical departments in an open research and deliberation process converged in a shared vision about the use of hypofractionation, both where a high level of scientific evidence supports its use and where the strength of the evidence is more limited. Following the literature review and the pooling of knowledge among experts with regard to the consensus-based recommendations on the use of hypofractionation in breast cancer, it is significant that 10 of the 14 questions posed yielded full agreement about the intervention. According our results, hypofractionation is appropriate in all patients aged 40 years or more with an indication for adjuvant external beam radiotherapy following breast-conserving surgery for invasive breast carcinoma and without nodal irradiation. The use of hypofractionation schemes in these cases is recommended regardless of other tumour characteristics, like size, histological grade, or molecular subtype; patient characteristics such as size or laterality of the breast; or treatment characteristics, including neoadjuvant or adjuvant chemotherapy, or need for a boost.

The degree of agreement did not meet our 80% cutoff for consensus in cases where available evidence was insufficient at the time of the review, that is, hypofractionation in patients younger than 40 years, receiving mastectomy, requiring irradiation of the lymph nodes, and with ductal carcinoma in situ. However, over half of the experts supported hypofractionation in all these situations.

Overall, our results are quite similar to the consensus reached by ASTRO [12] and reflected on the guidelines, despite the consideration of the age grouping in our survey and the inclusion of questions about the use of hypofractionation in mastectomized patients or in whom node irradiation is indicated.

Future lines of work, following the application of the consensus-based recommendations, will include an analysis of whether these contribute to changing the use of hypofractionation in breast cancer, standardising practice across the network of Catalan public hospitals. We will also analyse the use of hypofractionation where it is more controversial, for example in cases of immediate reconstruction with prosthesis or autologous graft, concomitant hypofractionated boost following intraoperative radiotherapy, or in the presence of autoimmune diseases.
